# Knowledge and Beliefs Surrounding Nalmefene Among Alabama Pharmacists: A Cross-Sectional Survey Informed by the Theory of Planned Behavior

**DOI:** 10.3390/pharmacy14060137

**Published:** 2026-09-17

**Authors:** Nicholas P. McCormick, Christian S. Flores, Olivia Aycock, Victoria Patterson, Francesca Mengel, Shannon Woods, Erin Blythe, Olivia Radzinski, Madison Holland, Melissa Sanders, Kathlyn Smith, Autumn Randles, Emma Tidmore, Bryson Grimsley, Anne Taylor, Brandy Davis, Lindsey Hohmann

**Affiliations:** Harrison College of Pharmacy, Auburn University, 2316 Walker Building, Auburn, AL 36849, USA

**Keywords:** nalmefene, opioid overdose, Alabama pharmacists, prevention, theory of planned behavior

## Abstract

The opioid epidemic continues to be a major public health crisis in the United States, and nalmefene has recently emerged as an alternative opioid overdose reversal agent. However, little is known about pharmacists’ awareness and beliefs regarding nalmefene use and distribution. Therefore, the purpose of this study was to assess Alabama pharmacists’ knowledge, perceptions, and behavioral intentions related to nalmefene. This study used a cross-sectional survey design. Practicing pharmacists in the state of Alabama were eligible to participate, and were recruited through the Alabama Board of Pharmacy email listserv. An anonymous online survey was distributed via email, and respondents were eligible to enter a lottery for one of five $100 electronic gift cards. The survey instrument was developed by the investigators, adapted from prior overdose reversal literature and informed by the Theory of Planned Behavior. Primary outcome measures included: knowledge of nalmefene (12 items); perceived barriers regarding nalmefene stocking and recommendations (19 items); and Theory of Planned Behavior concepts including attitudes (15 items), subjective norms (perceived social support) (six items), perceived behavioral control (confidence in ability to stock or recommend nalmefene) (11 items), and behavioral intentions surrounding nalmefene stocking and recommendations (13 items). Outcomes were measured via multiple-choice (objective knowledge) and Likert-type scale (1 = strongly disagree, 5 = strongly agree) questions. Differences in mean scale scores across pharmacy setting (pharmacists employed in inpatient versus outpatient settings), community pharmacy type (chain vs. independent), and geographic location (rural vs. urban) were analyzed using two-sided Mann–Whitney U tests for non-parametric data or *t*-tests for parametric data, as appropriate. Multiple linear regression analyses examined associations between TPB constructs and pharmacists’ intentions to stock or recommend nalmefene over the next six months. All data were analyzed using SPSS statistical software version 29 with an alpha of 0.05. There were 119 pharmacist respondents (*n* = 25 inpatient, *n* = 94 outpatient). The majority were female (68.1%), White (94.1%), and worked in community settings (35.3% independent, 10.9% corporate, 10.1% big-box, 5.0% grocery). Median age was 42 years (IQR: 36–51). Awareness of nalmefene was low: only 39.5% had heard of it and 5.9% had seen it in practice. Inpatient versus outpatient pharmacists reported higher subjective norms external to the workplace (median [IQR]: 3.00 [2.33, 3.58] vs. 2.33 [1.67, 3.00]; *p* = 0.004) as well as subjective knowledge (mean [SD]: 2.96 [0.94] vs. 2.55 [0.74]; *p* = 0.023), while outpatient versus inpatient pharmacists reported higher perceived barriers related to fiscal/logistical concerns (mean [SD]: 3.45 [0.59] vs. 3.07 [0.66]; *p* = 0.009). Among community pharmacists, stocking and procurement confidence was higher among independent versus chain pharmacists (3.75 [3.00–4.17] vs. 3.17 [2.67–3.92]; *p* = 0.026). Furthermore, external workplace norms among urban pharmacists were higher than among rural pharmacists (2.50 [2.00–3.00] vs. 2.33 [1.67, 2.67]; *p* = 0.027), while stocking and procurement confidence was higher among rural than urban pharmacists (3.83 [2.83–4.50] vs. 3.17 [2.67–4.00]; *p* = 0.034). In adjusted reduced regression models, perceived behavioral control regarding nalmefene stocking and procurement (B = 0.189, 95% CI: 0.049, 0.329; *p* = 0.009), subjective norms external to the workplace (B = 0.232, 95% CI: 0.047, 0.418; *p* = 0.015), and interprofessional recommendation attitudes (B = 0.237, 95% CI: 0.037, 0.437; *p* = 0.021) positively predicted nalmefene implementation intentions. These findings suggest practice-setting differences that could be further explored for optimal implementation of nalmefene distribution and access, and highlight the need for targeted education, interprofessional collaboration, procurement guidance, and workflow tools to improve pharmacist preparedness for nalmefene services.

## 1. Introduction

Opioid overdose remains a critical public health issue in the United States (U.S.). In 2023, there were over 105,000 persons in the U.S. that died from drug-involved overdoses including illicit or prescription drugs [1]. Although the national drug overdose death rate fell nearly 24% to 87,000 by the end of September 2024 [2], drug overdose still remains a leading cause of mortality for Americans aged 18–44 [3]. This landscape is further complicated by geographic disparities in overdose mortality and opioid use at the county and state levels, with higher rates of drug overdose deaths typically encountered in urban versus rural counties throughout the nation [4], primarily attributed to use of illicitly manufactured synthetic and semi-synthetic opioids (e.g., fentanyl, heroin) in urban areas versus misuse of natural and semi-synthetic prescription opioids (e.g., codeine, oxycodone, hydrocodone) in rural areas [4]. In the state of Alabama, which is largely rural but comprised of several dense urban clusters [5], geographic disparities in opioid overdose mortality are even more pronounced, with statewide rates (17.3 per 100,000 persons) exceeded the national average (16.0 per 100,000) in 2024 [6], and rates in excess of 45 per 100,000 in some urban hubs (e.g., Jefferson County) [7]. Given one of the highest rates of prescription opioid dispensing in the country (68.5 per 100,000 in Alabama versus 35.4 per 100,000 nationally) [8], and the growing threat of illicitly manufactured fentanyl across the state [9], strategies to reduce opioid overdose mortality across all regions of Alabama are crucial.

Naloxone, a short-acting opioid antagonist, has been the hallmark of overdose response efforts, specifically in the community setting. Take-home naloxone, available in injectable as well as ready-to-use nasal spray formulations, has been shown to increase bystander overdose response and reduce opioid overdose mortality [10,11]. However, the recent rise of highly potent, long-acting, illicit synthetic opioids (e.g., fentanyl) has created a pharmacological challenge for opioid overdose reversal. Thus, a singular, conventional opioid overdose reversal agent (naloxone) may no longer be sufficient. Accordingly, nalmefene has recently emerged as a promising alternative opioid antagonist. Similar to naloxone, nalmefene can rapidly (within 2–5 min) reverse the effects of opioid overdose, including respiratory depression, sedation, and low blood pressure [12,13,14]. In contrast, nalmefene possesses a longer half-life compared to naloxone (10.8 h versus 1–2 h for naloxone) [13,15,16,17], which may be beneficial for reversing overdoses caused by long-acting or highly potent synthetic opioids. Although a generic version of nalmefene was available for many years in an injection vial for intravenous administration, this dosage form was not routinely intended for layperson administration [14,16,18]. In May 2023, the U.S. Food and Drug Administration (FDA) approved the first commercially available ready-to-use prescription nalmefene nasal spray (Opvee^®^) for community opioid overdose response [12,13]. This was followed by the approval of a nalmefene auto-injector (Zurnai^®^) for intramuscular or subcutaneous administration in 2024, which became available on the market in mid-2025 [19,20]. As new opioid overdose reversal formulations become available for layperson administration, methods to increase patient access are increasingly important.

Pharmacists are qualified and well-positioned to increase access to opioid reversal agents through dispensing, counseling, and providing education. In fact, pharmacists dispensed more than 1.5 million naloxone prescriptions in 2024, highlighting the important role they play in community overdose prevention efforts [21]. Although several studies have evaluated pharmacists’ knowledge, attitudes, and dispensing practices related to naloxone [22,23,24], little is known about pharmacists’ awareness, perceptions, or willingness to recommend newly approved nalmefene formulations. Alabama represents a particularly important setting in which to examine nalmefene implementation given its high opioid dispensing rate [8], substantial rural population [5], and documented disparities in opioid overdose outcomes [6,7]. However, pharmacy-based naloxone access varies across pharmacy types and locations in Alabama, with corporately owned (chain) pharmacies more likely to stock naloxone versus their independently owned (independent) counterparts, and longer wait times for pharmacies to procure naloxone in rural versus urban locations [25]. Given these existing disparities in naloxone access [25], understanding pharmacists’ perceptions surrounding community-based nalmefene services across a variety of pharmacy settings will help to identify actionable implementation barriers and facilitators and lay the groundwork for future educational, practice, and policy interventions. Therefore, the purpose of this study was to assess Alabama pharmacists’ awareness, knowledge, beliefs, and intentions surrounding stocking, selling, and recommending nalmefene and to identify factors that may influence its future implementation across pharmacy types and locations.

## 2. Materials and Methods

### 2.1. Study Design, Participants, and Recruitment

The study employed a cross-sectional survey design. Individuals were eligible to participate if they were ≥19 years old, licensed as a pharmacist in the state of Alabama, and employed in any pharmacy setting (e.g., inpatient, ambulatory care, community). Participants were recruited via the Alabama Board of Pharmacy email listserv, which includes all licensed pharmacists practicing within the state, with an original email invitation followed by three reminder emails at 2- to 3-week intervals over a 2-month recruitment window from February to April 2025. Recruitment emails included a brief study overview and link to the online survey. After reviewing the information letter on the first page of the survey, individuals electronically provided consent to participate before proceeding to the survey questions. This recruitment strategy was utilized to maximize reach across both rural and urban regions of Alabama, as well as a variety of pharmacy practice settings. To incentivize participation, respondents who completed the survey were eligible to enter a lottery for one of five $100 electronic gift cards.

### 2.2. Sample Size Calculation

Assuming a medium effect size of f^2^ = 0.21 [26,27] and α = 0.05, a minimum sample size of 114 was determined to be sufficient to evaluate predictors of intention to stock/recommend nalmefene (the primary outcome of interest) via multiple linear regression with 80% power. Power calculations were conducted using G*Power software version 3.1.9.7 (Heinrich Heine Universität, Düsseldorf, Germany) [27,28].

### 2.3. Data Collection and Measures

Data were collected via an anonymous online survey distributed via email. The survey instrument was developed by the investigators, with measures informed by the Theory of Planned Behavior (TPB), which proposes that behavioral intentions are shaped by attitudes, subjective norms (SN), and perceived behavioral control (PBC) [29]. The survey instrument was pre-tested for face and content validity by content experts at the corresponding author’s institution (n = 1), and item wording was revised according to feedback prior to survey distribution. Primary outcome measures included: objective (5 items) and subjective (7 items) knowledge of nalmefene; perceived barriers regarding nalmefene stocking and recommendations (19 items); and TPB concepts including attitudes (15 items), subjective norms (perceived social support) (6 items), perceived behavioral control (confidence in ability to stock or recommend nalmefene) (11 items), and behavioral intentions surrounding nalmefene service implementation in the next 6 months (13 items). Outcomes were measured via multiple-choice questions (objective knowledge) and Likert-type scales (1 = strongly disagree, 5 = strongly agree), with items adapted from prior opioid overdose reversal literature [13,30,31,32,33]. Specifically, objective knowledge questions were informed by literature available from the FDA [13,31,32], manufacturers [33], and Thompson et al. [30]. Attitude, perceived barrier, and intention items were further informed by Thompson [33], Rudolph [34], and Hohmann [24] and colleagues.

Secondarily, pharmacy setting (community, hospital, clinic, etc.), community pharmacy type (corporately owned freestanding store, grocery, big-box, or independently owned), geographic location (Alabama county), position within the pharmacy (manager, part-time, full-time, etc.), personal or professional experience with OUD (yes/no), and the primary patient population served by the pharmacy (age and socioeconomic status) were assessed via multiple-choice. Participant demographic characteristics (age, race, ethnicity, and gender) were collected via self-report. The full survey instrument is available in Supplemental File S1.

### 2.4. Data Analysis

Primary and secondary outcomes were characterized using descriptive statistics. The percentage of objective knowledge questions answered correctly were averaged across respondents to create an overall objective knowledge score. Likert-type items were likewise summed and averaged across respondents to create overall average subjective knowledge, perceived barriers, attitudes, SN, PBC, and intention scale scores. Likert-type items were reverse coded as necessary prior to the calculation of average scale scores, such that higher values indicated more of a construct (e.g., more perceived barriers, more positive attitudes, greater social support, higher intentions). Missing data at the item-level was excluded from analysis, and respondents with >20% of items incomplete were removed from the dataset. Furthermore, construct validity of subjective knowledge, perceived barriers, attitudes, SN, PBC, and intention scales was examined using exploratory factor analysis (EFA) with principal axis factoring and direct oblimin rotation. Although the Guttman–Kaiser criterion conventionally retains factors with eigenvalues greater than 1.0 [35,36], this rule may overextract dimensions and yield superfluous, less parsimonious solutions [37,38]; therefore, a conservative study-wide initial-eigenvalue threshold of ≥1.60 was applied in conjunction with scree-plot inspection, theoretical coherence, factor interpretability, and parsimony, consistent with methodological guidance to consider multiple statistical and substantive criteria when determining factor (sub-scale) retention [39]. Absolute pattern-matrix loadings ≥0.40 were considered salient, and items without a salient loading on any retained factor were excluded from final scale scoring [40]. Bartlett’s test of sphericity and Kaiser–Meyer–Olkin (KMO) statistics were used to assess the appropriateness of the data for factor analysis. Internal consistency of survey scales and sub-scales was assessed using the Cronbach’s alpha (α) statistic, with a threshold of α ≥ 0.7 indicating acceptable reliability [41]. The final scale and sub-scale scores were calculated as the mean of the retained items after reverse coding, as applicable.

Differences in mean scale and sub-scale scores across pharmacy setting (dichotomized as pharmacists employed in inpatient or institutional vs. outpatient settings), community pharmacy type (dichotomized as chain [corporately owned freestanding store, grocery, or big-box] vs. independent), geographic location (dichotomized as rural vs. urban), personal or professional experience with OUD (yes/no), and position within the pharmacy (dichotomized as managerial vs. non-managerial) were analyzed using two-sided *t*-tests for normally distributed data or Mann–Whitney U tests for non-normally distributed data, as assessed by the Shapiro–Wilk test. Cohen’s *d* (|d|: 0.2 = small, 0.5 = medium, 0.8 = large effect) [42,43] and rank-biserial correlation coefficients (*r_rb_* = |Z|/√N: 0.1 = small, 0.3 = medium, 0.5 = large effect) [43,44] were utilized as measures of effect size for parametric and non-parametric tests, respectively. Dichotomization of sociodemographic variables was performed to retain statistical power, given limited sample sizes within several sociodemographic categories. Geographic location was categorized as rural or urban according to the Alabama Rural Health Association county-level designations [45].

Additionally, multiple linear regression analyses examined predictors of pharmacists’ intentions to stock or recommend nalmefene over the next six months. In the primary theory-driven (“full”) model, mean intention scale score was regressed on mean scale or sub-scale scores for objective knowledge, subjective knowledge, perceived barriers, attitudes, SN, and PBC. Two versions of the full model were analyzed: an unadjusted model including only the primary predictors of interest; and an adjusted model controlling for sociodemographic characteristics of age, gender (female vs. male), race (American Indian or Alaska Native, Asian, Black or African American, Native Hawaiian or Other Pacific Islander vs. White), pharmacy setting (inpatient or institutional vs. outpatient), geographic location (rural vs. urban), position within the pharmacy (managerial vs. non-managerial), personal or professional experience with OUD (yes/no), the age of the primary patient population served by the pharmacy (children/adolescents, younger adults, older adults, or a mix of ages), and whether the pharmacy served indigent or low-income patients (yes/no). As a secondary exploratory analysis, a reduced model was evaluated using stepwise variable selection to identify a more parsimonious set of predictors associated with intention to stock or recommend nalmefene. Stepwise variable selection procedures used probability-of-F criteria of *p* ≤ 0.05 for variable entry and *p* ≥ 0.10 for variable removal, as applicable. Both unadjusted and adjusted versions of the reduced model were examined, controlling for the aforementioned sociodemographic covariates.

Prior to conducting multiple linear regression analyses, regression assumptions were evaluated. Multicollinearity was assessed using variance inflation factors (VIFs) and tolerance statistics. Independence of errors was evaluated using the Durbin–Watson statistics, and potentially influential observations were assessed using Cook’s distance. Multivariate normality of residuals was evaluated through visual inspection of histograms and normal probability (P-P) plots of standardized residuals. Homoscedasticity was assessed through visual inspection of scatterplots of standardized residuals versus standardized predicted values. All data were analyzed using SPSS statistical software version 29 (IBM Corp., Armonk, NY, USA) with an alpha of 0.05.

### 2.5. Ethics Approval

All study procedures were approved by the Institutional Review Board (IRB) at the corresponding author’s institution (Protocol #STUDY00000368), and informed consent to participate was provided by all respondents.

## 3. Results

### 3.1. Participant Characteristics

Of the 5281 individuals contacted, 142 clicked on the survey (2.7% response rate) and 129 consented to participate and submitted the survey. Of these, a total of 119 pharmacists completed at least 80% of the survey items and were retained in the final dataset (83.8% completion rate). The median participant age was 42 years (IQR 36–51) (Table 1). Most respondents identified as female (68.1%) and White (94.1%). Over half (61.3%) reported non-managerial roles, while 38.7% held managerial or administrative positions. Pharmacy settings were diverse: 35.3% worked in independently owned community pharmacies, 16.8% in hospital inpatient settings, 10.9% in corporately owned community pharmacies, with smaller proportions practicing in grocery, big-box, specialty pharmacies, or clinic-based settings. A majority practiced in outpatient (79.0%) versus inpatient or institutional (21.0%) locations, and were in urban areas (68.9%). Approximately 27% reported serving predominantly low-income or indigent populations. Regarding opioid use disorder (OUD) experiences, 42.0% reported being personally and/or professionally affected. Furthermore, awareness of nalmefene was generally low, with 56.3% having never heard of nalmefene prior to the survey, and only 5.9% having seen nalmefene in practice.

### 3.2. Objective and Subjective Knowledge

Objective knowledge about nalmefene was limited, with a mean (SD) overall objective knowledge score of 49.47% (17.86) (Table 2). While 66.4% correctly identified nalmefene as indicated for opioid overdose reversal, only 24.4% knew it was available as a nasal spray, and 30.3% correctly identified its onset of action (2–5 min). Knowledge of wholesale cost was particularly low, with only 21.8% selecting the correct price range for the commercially available nasal spray. See Supplemental File S2 (Supplemental Tables) for item-level analyses.

In terms of subjective knowledge, exploratory factor analysis indicated five of the seven subjective knowledge items loaded on a single factor (Cronbach’s alpha = 0.798) (Bartlett’s Test of Sphericity: *p* < 0.001; KMO = 0.791) (Table 2 and Supplemental File S2). Overall, subjective knowledge about nalmefene was fairly low (mean [SD]: 2.63 [0.80]). In particular, 34.5% agreed or strongly agreed that they knew how to counsel a patient about nalmefene, 43.2% that they knew the differences between nalmefene and naloxone, and 80.7% that they needed more information about nalmefene.

The mean composite objective knowledge score did not differ significantly across pharmacy locations, settings, community pharmacy types, personal/professional experience with OUD, or job roles (Table 3, Table 4, Table 5, Table 6 and Table 7). In contrast, mean (SD) subjective knowledge was higher among those who reported versus did not report personal or professional experience with OUD (2.88 [0.85] vs. 2.46 [0.73]; *d* = 0.526, *p* = 0.006) and among inpatient versus outpatient pharmacists (2.96 [0.94] vs. 2.55 [0.74]; *d* = 0.519, *p* = 0.023).

### 3.3. Attitudes

Exploratory factor analysis indicated that attitudes were composed of two factors: clinical attitudes (Cronbach’s alpha = 0.857); and interprofessional recommendation attitudes (Cronbach’s alpha = 0.877) (Bartlett’s Test of Sphericity: *p* < 0.001; KMO = 0.842) (Table 2 and Supplemental File S2). Overall, attitudes about nalmefene were positive (mean [SD] scale score: 3.69 [0.53]). Clinical attitudes were slightly higher (3.79 [0.56]) than interprofessional recommendation attitudes (3.49 [0.65]). In particular, the importance of pharmacist knowledge about nalmefene was rated highly, with 95.7% agreeing or strongly agreeing, and 82.9% supporting nalmefene as a good option for patients at risk of opioid overdose. Interestingly, while 71.8% indicated that they would recommend nalmefene to another healthcare provider, fewer (61.6%) endorsed recommending nalmefene to law enforcement officers. Pharmacists in managerial/administrative roles reported significantly more favorable attitudes toward recommending nalmefene to interprofessional colleagues (mean [SD]; 3.65 [0.47]) compared with non-managerial pharmacists (3.39 [0.73]; *r_rb_* = 0.209, *p* = 0.023) (Table 3, Table 4, Table 5, Table 6 and Table 7).

### 3.4. Subjective Norms

Exploratory factor analysis indicated that subjective norms were composed of two factors: external to the workplace norms (Cronbach’s alpha = 0.817); and internal workplace norms (Cronbach’s alpha = 0.816) (Bartlett’s Test of Sphericity: *p* < 0.001; KMO = 0.734) (Table 2 and Supplemental File S2). Overall, the mean (SD) subjective norms score was moderately low (2.87 [0.59]). Norms external to the workplace were lower (2.49 [0.76]) compared with internal workplace norms (3.26 [0.71]). In particular, 64.7% of pharmacists disagreed or strongly disagreed that nalmefene was being prescribed in their area and 75.0% were neutral about patient openness to using nalmefene. Median (IQR) subjective norms were higher overall among inpatient versus outpatient pharmacy settings (3.00 [2.54, 3.79] vs. 2.83 [2.50, 3.00]; *r_rb_* = 0.200, *p* = 0.029) (Table 3, Table 4, Table 5, Table 6 and Table 7). At the sub-scale level, norms external to the workplace were higher among inpatient versus outpatient pharmacy settings (3.00 [2.33, 3.58] vs. 2.33 [1.67, 3.00]; *r_rb_* = 0.264, *p* = 0.004) and among urban versus rural pharmacists (2.50 [2.00–3.00] vs. 2.33 [1.67, 2.67], *r_rb_* = 0.202, *p* = 0.027).

### 3.5. Perceived Behavioral Control

Exploratory factor analysis indicated that perceived behavioral control was composed of two factors: nalmefene stocking and procurement (Cronbach’s alpha = 0.887); and nalmefene counseling confidence (Cronbach’s alpha = 0.915) (Bartlett’s Test of Sphericity: *p* < 0.001; KMO = 0.822) (Table 2 and Supplemental File S2). Mean (SD) perceived behavioral control was positive overall at 3.35 (0.78). Stocking and procurement confidence was slightly higher (3.35 [0.93]) than nalmefene counseling confidence (3.29 [0.92]). Half of the pharmacists (50.0%) disagreed or strongly disagreed that stocking nalmefene in their workplace was outside of their control and 46.5% agreed or strongly agreed that they felt confident in their ability to counsel patients on nalmefene. Of note, mean (SD) perceived behavioral control was higher overall among pharmacists in managerial versus non-managerial roles (3.63 [0.70] vs. 3.14 [0.74]; *d* = 0.670, *p* < 0.001) (Table 3, Table 4, Table 5, Table 6 and Table 7). At the sub-scale level, median (IQR) stocking and procurement confidence was also greater among those in managerial versus non-managerial positions (4.00 [3.17, 4.50] vs. 3.00 [2.50, 3.67]; *r_rb_* = 0.400, *p* < 0.001) and among independent versus chain community pharmacy types (3.75 [3.00–4.17] vs. 3.17 [2.67, 3.92]; *r_rb_* = 0.260, *p* = 0.026), as well as among rural pharmacists compared with their urban counterparts (3.83 [2.83–4.50] vs. 3.17 [2.67–4.00]; *r_rb_* = 0.195, *p* = 0.034). In addition, pharmacists with personal or professional experience with opioid use disorder also reported greater mean (SD) perceived behavioral control overall (3.49 [0.77] vs. 3.19 [0.73]; *d* = 0.400, *p* = 0.037) and greater median (IQR) counseling confidence (3.80 [2.90, 4.00] vs. 3.40 [2.40, 3.90]); *r_rb_* = 0.192, *p* = 0.036) than those without such experience.

### 3.6. Perceived Barriers

Exploratory factor analysis indicated that perceived barriers was composed of two factors: fiscal and logistical barriers (Cronbach’s alpha = 0.831); and ethical and stakeholder barriers (Cronbach’s alpha = 0.817) (Bartlett’s Test of Sphericity: *p* < 0.001; KMO = 0.802.) (Table 2 and Supplemental File S2). Overall, mean (SD) perceived barriers were fairly neutral (2.98 [0.51]), although fiscal and logistical barriers (3.37 [0.62]) were rated higher than ethical and stakeholder barriers (2.53 [0.57]). In particular, 44.1% of pharmacists reported concern that patients would not be able to afford nalmefene, and 45% identified reimbursement for nalmefene dispensing as a barrier. About 23% were unsure how to approach patients about the need for nalmefene, and 17.1% identified lack of management support as a barrier to pharmacy-based nalmefene provision. A small percentage (4.5%) reported moral/ethical concerns with dispensing nalmefene. Mean (SD) perceived barriers were greater among outpatient pharmacists compared with inpatient pharmacists overall (3.05 [0.51 vs. 2.75 [0.56]; *d* = 0.567, *p* = 0.017) and related to fiscal/logistical barriers (3.45 [0.59] vs. 3.07 [0.66]; *d* = 0.628, *p* = 0.009) (Table 3, Table 4, Table 5, Table 6 and Table 7).

### 3.7. Intentions

Exploratory factor analysis indicated that twelve of the 13 intention items loaded on a single factor (Cronbach’s alpha = 0.950) (Bartlett’s Test of Sphericity: *p* < 0.001; KMO = 0.893) (Table 2 and Supplemental File S2). Overall, mean (SD) intention to implement nalmefene services in the next 6 months was fairly neutral (3.06 [0.69]). In particular, 17.4% reported they were likely to stock nalmefene in their workplace, 32.2% to counsel patients regarding nalmefene, and 37.6% to recommend it to patients prescribed ≥50 MME opioids. Willingness to recommend nalmefene to law enforcement (30.2%) was slightly lower than to other healthcare providers (35.8%). About one-quarter (24.8%) intended to stock both nalmefene and naloxone. Intentions did not differ significantly across pharmacy locations, settings, community pharmacy types, personal/professional experience with OUD, or job roles (Table 3, Table 4, Table 5, Table 6 and Table 7).

Additionally, regression analyses examined predictors of pharmacists’ intentions to implement nalmefene services over the next six months. In the adjusted full model (Table 8), perceived behavioral control regarding nalmefene stocking and procurement was positively associated with implementation intentions (B = 0.170, 95% CI: 0.002, 0.338; *p* = 0.047), whereas inpatient practice was associated with lower intention scores relative to outpatient practice (B = −0.358, 95% CI: −0.718, −0.002; *p* = 0.048). In the adjusted reduced model (Table 9), perceived behavioral control regarding nalmefene stocking and procurement remained a significant positive predictor (B = 0.189, 95% CI: 0.049, 0.329; *p* = 0.009), in addition to subjective norms external to the workplace (B = 0.232, 95% CI: 0.047, 0.418; *p* = 0.015) and interprofessional recommendation attitudes (B = 0.237, 95% CI: 0.037, 0.437; *p* = 0.021). Inpatient pharmacy setting remained a significant negative predictor (B = −0.366, 95% CI: −0.668, −0.065; *p* = 0.018). Regression diagnostic testing indicated that model assumptions were adequately met across all models (Table 8 and Table 9).

## 4. Discussion

To the authors’ knowledge, this is among the first studies to evaluate pharmacists’ awareness, perceptions, and implementation intentions regarding nalmefene following approval of the first community-administered formulations. Despite generally favorable attitudes toward nalmefene and pharmacist involvement in overdose prevention, awareness and knowledge of nalmefene were limited, perceived structural barriers were high, implementation intentions were only moderate, and perceived behavioral control appeared to play a prominent role in shaping future adoption.

Pharmacists demonstrated particularly low objective knowledge of nalmefene. Although approximately two-thirds correctly identified its indication for opioid overdose reversal, fewer than one-quarter recognized its intranasal route of administration, and fewer than one-third were aware of its onset of action. The weakest areas of performance involved pharmacokinetic properties—particularly duration of effect—and cost awareness, suggesting ongoing uncertainty regarding how nalmefene differs pharmacologically and commercially from naloxone. This knowledge deficit is consistent with prior studies of naloxone among pharmacists; for example, Nielsen et al. reported a mean score of 1.8 out of 5 (~36%) regarding naloxone administration understanding among Australian pharmacists [46]. Additionally, the survey was conducted relatively soon after approval of community-administered nalmefene formulations. Thus, limited awareness and knowledge (both objective and subjective) may reflect the novelty of nalmefene, necessitating future studies to assess nalmefene awareness and knowledge as new formulations become more widely available over time. Given these factors, targeted educational interventions are warranted. Effective strategies may include continuing education modules, structured in-service training, manufacturer-provided fact sheets, and integration of nalmefene content into pharmacy curricula. For example, a multi-phase naloxone training program incorporating didactic, team- and case-based learning in second-year pharmacy students achieved significant improvements in knowledge and confidence [47].

Despite low knowledge and awareness, attitudes toward nalmefene were generally favorable and did not differ by geography or practice type. These findings are consistent with prior research by Burstein et al. that found generally positive attitudes toward pharmacy-based naloxone [48], while O’Brien et al. similarly observed high acceptance rates for naloxone dispensing roles among community pharmacists (~90%) [49]. Collectively, this suggests a sustained professional openness to overdose reversal initiatives. That said, attitudes towards interprofessional recommendations of nalmefene tended to be lower compared to attitudes surrounding nalmefene’s clinical utility. This may indicate that pharmacists recognize nalmefene’s potential benefits but remain hesitant to actively recommend it to other healthcare professionals or community stakeholders due to limited familiarity with newly marketed formulations or uncertainty regarding its place relative to naloxone. However, managerial-role pharmacists held more favorable interprofessional recommendation attitudes, possibly reflecting greater involvement in formulary decisions, policy implementation or leadership responsibilities. Given this discrepancy, future initiatives may wish to obtain management support (e.g., involving executive boards in the implementation process or obtaining written agreements from key leadership personnel) and identify pharmacist champions to enhance organizational adoption and broaden implementation of nalmefene services [50,51].

Subjective norms—particularly external norms—were moderate. Pharmacists reported limited external support, with many disagreeing that nalmefene was being prescribed locally, and approximately 75% remaining neutral regarding patient openness. Notably, external subjective norms appeared less favorable than attitudes, suggesting that pharmacists were generally receptive to nalmefene but perceived less support for its adoption from prescribers, patients, and the broader healthcare environment. Rural pharmacists also perceived weaker external normative support than their urban counterparts. Given the recent introduction of community-administered nalmefene formulations, this finding may reflect fewer opportunities in rural areas to observe prescribing, dispensing, and patient utilization, thereby limiting perceptions of community acceptance and professional endorsement. In contrast, internal workplace norms appeared stronger across all pharmacy settings, types, and locations. This pattern aligns with prior research on naloxone implementation, where regional policy climate and peer behaviors influenced adoption. For example, Laing et al. found that Australian pharmacies offering needle/syringe programs and reporting greater comfort in overdose counseling were more likely to stock naloxone [52]. The comparatively weak external normative pressure in the current study suggests that greater clinician endorsement, policy support, and visible community uptake might strengthen pharmacists’ normative motivation and accelerate nalmefene integration into practice. Indeed, strategies to overcome barriers in the external environment are key to enhancing innovation implementation [53,54]. Future implementation efforts should therefore focus on increasing the visibility of nalmefene use through interprofessional education, prescriber–pharmacist collaboration, and dissemination of local success stories, particularly in rural communities where perceptions of external support were less favorable.

Perceived behavioral control was positive overall. However, pharmacists felt more confident about nalmefene procurement than counseling. Similarly, managerial pharmacists reported higher perceived behavioral control overall and regarding procurement confidence. This may be due to managers having authority over inventory decisions and greater familiarity with wholesaler ordering systems, and staff pharmacists perceiving less autonomy. Additionally, in contrast to previous research that found longer wait times for pharmacies to procure naloxone in rural versus urban locations as well as greater likelihood of stocking naloxone in chain versus independent pharmacies [25], the current study reported greater nalmefene stocking confidence among rural and independent pharmacists. This may reflect the greater operational autonomy often afforded to independent pharmacies, which are frequently located in rural communities, where pharmacists typically have more direct influence over purchasing decisions, inventory management, and service implementation. Conversely, chain pharmacists may face additional organizational approval processes, standardized formularies, or corporate policies that limit their perceived control over stocking decisions. Taken all together, efforts to enhance staff pharmacists’ self-efficacy—through procurement guidance (e.g., procurement checklists), workflow integration (e.g., service process maps), peer modeling (e.g., testimonials from successful pharmacies), and practical counseling training (e.g., videos demonstrating communication approaches)—may further strengthen implementation readiness across all locations and pharmacy types, particularly urban and chain pharmacies where confidence was the lowest.

Although perceived barriers to nalmefene services were neutral overall, perceived fiscal and logistical barriers exceeded moral or ethical barriers. Indeed, only about 4.5% reported moral or ethical concerns, while most concerns centered on practical implementation issues such as product cost, reimbursement, and procurement. These findings suggest that resistance to nalmefene adoption is unlikely to stem from philosophical opposition to overdose prevention efforts, but rather from uncertainty regarding how to successfully integrate the product into routine practice. This uncertainty represents an actionable barrier that can be targeted in future educational initiatives. System-level fiscal and logistical barriers were also higher among pharmacists employed in the outpatient setting compared to their inpatient or institutional counterparts. This may be explained by the fact that outpatient pharmacists are more directly involved in medication purchasing, inventory management, third-party reimbursement, and patient cost discussions, making them particularly aware of the financial and operational challenges associated with introducing new products. In contrast, inpatient and institutional formularies often have centralized purchasing mechanisms and established medication procurement processes that may lessen pharmacists’ perceptions of these barriers. These findings are consistent with prior naloxone research, which has identified cost, reimbursement, workflow burden, and operational constraints as important barriers to pharmacist-led overdose prevention services [25,34]. Thus, consistent with implementation science frameworks emphasizing both individual and organizational determinants of practice change [53,54], educational initiatives alone may be insufficient to advance nalmefene adoption and should be coupled with system-level supports such as reimbursement mechanisms, consistent supply chains, formulary inclusion, and organizational policies that facilitate implementation within routine pharmacy practice.

Additionally, although pharmacists generally supported nalmefene, intention to stock or recommend the medication within the next six months was relatively neutral. This gap between favorable attitudes and implementation intentions suggests that support for nalmefene alone may be insufficient to drive adoption when knowledge deficits, perceived barriers, and limited confidence remain. Indeed, consistent with the Theory of Planned Behavior [29], pharmacists in the current study who reported greater perceived behavioral control regarding nalmefene stocking and procurement, stronger external subjective norms, and more favorable interprofessional recommendation attitudes demonstrated stronger implementation intentions in regression analyses. Therefore, to bridge the attitude–implementation gap, future interventions may wish to focus on increasing pharmacists’ confidence regarding nalmefene procurement, enhancing community awareness of nalmefene, and promoting interdisciplinary collaboration. Potential strategies may include procurement guidance, workflow integration tools, and targeted educational programming, as prior naloxone studies have shown that training interventions can improve pharmacist knowledge, confidence, and implementation of overdose prevention services [24,47]. Similarly, increasing visibility of nalmefene via public health campaigns and interprofessional education may strengthen normative support for implementation, promoting a culture of comfort shown to influence adoption of overdose prevention services [52]. Of note, inpatient practice predicted lower nalmefene implementation intentions in regression analyses. This finding may reflect differences in perceived professional roles across practice settings. Community pharmacists more routinely engage in outpatient dispensing, patient counseling, and take-home overdose prevention activities, whereas inpatient pharmacists may perceive nalmefene distribution as occurring outside their primary responsibilities or workflow. To enhance adoption of nalmefene within inpatient and institutional settings, future educational initiatives may wish to highlight potential inpatient and transitions-of-care applications of nalmefene, including emergency medicine use, long-term care facility use, and discharge counseling for patients receiving opioid therapy.

### Limitations

The study design has several limitations. As a cross-sectional survey, causality cannot be established, and observed associations should be interpreted cautiously. Although content and face validity of the survey instrument were pre-tested prior to survey distribution, the pre-test sample size was low (n = 1); future studies may wish to utilize a broader pre-test sampling frame to recruit a larger pool of content experts. Further, the survey response rate was low (2.7%), potentially leading to non-response bias. Selection bias cannot be discounted, as respondents voluntarily completed the survey and may have been more interested in opioid-related topics than non-respondents. Social desirability bias may have resulted in inflation of favorable attitude or intention responses. In particular, given that over half of respondents had not heard of nalmefene before taking the survey, positive attitudes and intentions regarding nalmefene may have been influenced by educational content embedded in the survey and should be interpreted cautiously. Additionally, all measures were self-reported and may not accurately reflect real-world nalmefene stocking, dispensing, or recommendation practices. Similarly, the Theory of Planned Behavior measures intentions, not actual behavior, and willingness to stock or recommend nalmefene may not translate into real-world implementation. Generalizability was limited to Alabama pharmacists, and findings may not extend to other states or regions. Furthermore, some subgroup analyses may have been underpowered due to limited sample sizes within certain practice settings (e.g., inpatient setting, managerial role) and demographic categories (e.g., rural location). Finally, the study was conducted shortly after approval of community-administered nalmefene formulations, and the evolving market environment may have contributed to low awareness, uncertainty regarding clinical use, perceived fiscal and logistical barriers, and only moderate implementation intentions. Future studies should reassess knowledge, perceived barriers, and implementation intentions and behaviors after new nalmefene formulations become more established on the market.

## 5. Conclusions

This study provides some of the first insights into pharmacists’ awareness, perceptions, and implementation intentions regarding nalmefene following the introduction of community-administered formulations. Although Alabama pharmacists generally viewed nalmefene favorably, awareness and knowledge were limited. Perceived behavioral control regarding nalmefene stocking and procurement, external subjective norms, and interprofessional recommendation attitudes emerged as important predictors of implementation intentions, while perceived barriers regarding cost and other logistical challenges remained prevalent. These findings suggest that structural and implementation-related barriers may represent greater obstacles to nalmefene adoption than attitudinal resistance. Consequently, efforts focused on pharmacist education, reimbursement support, supply-chain accessibility, prescriber engagement, and implementation readiness may be more important than attitude change alone in promoting nalmefene integration into pharmacy practice. Strengthening these supports may help expand pharmacist participation in overdose prevention efforts and improve access to the expanding market of opioid overdose reversal agents.

## Figures and Tables

**Table 1:** Participant and pharmacy characteristics (*n* = 119).

Characteristic	n (%)
Gender	
Female	81 (68.1)
Male	38 (31.9)
Race ^a^	
White	112 (94.1)
Asian	4 (3.4)
Black or African American	3 (2.5)
American Indian or Alaskan Native	1 (0.8)
Native Hawaiian or Other Pacific Islander	-
Hispanic or Latino(a)	
Yes	-
No	119 (100.0)
Position at Pharmacy	
Pharmacy manager	37 (31.1)
Full-time staff pharmacist	31 (26.1)
Part-time staff pharmacist	17 (14.3)
Clinical pharmacist	14 (11.8)
Floating pharmacist	11 (9.2)
Other	5 (4.2)
Assistant pharmacy manager	4 (3.4)
Managerial/Administrative Pharmacy Position Status	
Non-managerial	73 (61.3)
Managerial/Administrative	46 (38.7)
Been Personally and/or Professionally Affected by Opioid use Disorder (OUD)	
No	69 (58.0)
Yes	50 (42.0)
Ever Heard of Nalmefene	
No	67 (56.3)
Yes	47 (39.5)
Unsure	5 (4.2)
Ever Seen Nalmefene in Practice	
No	108 (90.8)
Yes	7 (5.9)
Unsure	4 (3.4)
Ever Been Asked about Nalmefene in Professional Capacity	
No	107 (89.9)
Yes	12 (10.1)
If Asked About Nalmefene, Who Asked ^a^	
Doctor	7 (58.3)
Another pharmacist	6 (50.0)
Other	4 (33.3)
Patient	2 (16.7)
Caregiver	-
Law Enforcement	-
Pharmacy Patient Population	
A mix of younger adults, older adults, and/or children	92 (77.3)
Primarily older adult patients (e.g., ≥65 y.o.)	19 (16.0)
Primarily younger adult patients (e.g., <65 y.o.)	5 (4.2)
Primarily children and adolescents	2 (1.7)
Missing	1 (0.8)
Pharmacy Serves Indigent and/or Low-Income Patients	
No	86 (72.3)
Yes	32 (26.9)
Missing	1 (0.8)
Pharmacy/Workplace Setting	
Community: Independently owned pharmacy	42 (35.3)
Hospital: Inpatient pharmacy	20 (16.8)
Other	14 (11.8)
Community: Corporately owned pharmacy	13 (10.9)
Community: Big-box store	12 (10.1)
Community: Grocery store	6 (5.0)
Specialty pharmacy	5 (4.2)
Clinic	4 (3.4)
Hospital: Outpatient pharmacy	3 (2.5)
Pharmacy/Workplace Inpatient-Outpatient Designation ^b^	
Outpatient	94 (79.0)
Inpatient or Institutional	25 (21.0)
Community Pharmacy Type ^c^	
Chain	31 (42.5)
Independent	42 (57.5)
Pharmacy Rural-Urban Designation ^d^	
Urban	82 (68.9)
Rural	36 (30.3)
Missing	1 (0.8)
**Characteristic**	**Median (IQR ^e^)**
Participant Age	42.0 (36.0, 51.0)

^a^ Total may add up to more than 100% as participants were asked to “Select All That Apply.”; ^b^ Outpatient settings incorporated community, specialty pharmacy, clinic, and hospital outpatient settings. Responses reported as “Other” were categorized as an inpatient or outpatient setting, according to participants’ free-text responses. For example, “compounding pharmacy” was categorized as an outpatient setting, and “long-term care” was categorized as an inpatient (institutional) setting. ^c^ Community pharmacy types were categorized as chain (corporately owned freestanding store, grocery, or big-box) or independent (independently owned). ^d^ Alabama Rural Health Association county-level rural-urban designations were used to determine rurality. ^e^ Interquartile range (IQR) is defined as the 25th and 75th percentiles.

**Table 2:** Characterization of knowledge, perceived barriers, and Theory of Planned Behavior (TPB) constructs (*n* = 119).

TPB Scale	Mean (SD)	Median (IQR)	Cronbach’s α	Shapiro–Wilk Test Statistic
Objective Nalmefene Knowledge Score ^a^	49.47 (17.86)	50.00 (37.50, 62.50)	-	0.923 ***
Subjective Nalmefene Knowledge Scale	2.63 (0.80)	2.60 (2.15, 3.20)	0.798	0.985
Attitudes Toward Nalmefene Scale	3.69 (0.53)	3.75 (3.35, 4.00)	0.891	0.986
Clinical Attitudes Toward Nalmefene	3.79 (0.56)	3.75 (3.50, 4.13)	0.857	0.921 ***
Interprofessional Recommendation Attitudes	3.49 (0.65)	3.50 (3.00, 4.00)	0.877	0.956 **
Subjective Norms Regarding Nalmefene Scale	2.87 (0.59)	2.83 (2.50, 3.17)	0.785	0.968 *
External to Workplace Subjective Norms	2.49 (0.76)	2.33 (1.75, 3.00)	0.817	0.948 ***
Internal Workplace Subjective Norms	3.26 (0.71)	3.33 (3.00, 3.67)	0.816	0.965 **
Perceived Behavioral Control Regarding Nalmefene Scale	3.35 (0.78)	3.41 (2.91, 3.82)	0.883	0.983
Nalmefene Stocking and Procurement	3.35 (0.93)	3.17 (2.67, 4.00)	0.887	0.972 *
Nalmefene Counseling Confidence	3.29 (0.92)	3.40 (2.40, 4.00)	0.915	0.952 ***
Barriers Regarding Nalmefene Scale	2.98 (0.51)	3.00 (2.65, 3.35)	0.869	0.979
Fiscal and Logistical Barriers	3.37 (0.62)	3.33 (3.00, 3.78)	0.831	0.983
Ethical and Stakeholder Barriers	2.53 (0.57)	2.50 (2.22, 3.00)	0.817	0.983
Nalmefene Services Intentions Scale	3.06 (0.69)	3.08 (2.88, 3.50)	0.950	0.943 ***

*** *p* < 0.001, ** *p* < 0.01, * *p* < 0.05; ^a^ Percent of questions answered correctly.

**Table 3:** Comparison of rural and urban pharmacies (*n* = 119).

Scale	Pharmacy Rural–Urban Designation		Effect Size ^c,d^	*p*-Value
	Rural *n* = 36	Urban *n* = 82		
	Mean (SD) ^a^ or Median (IQR) ^b^	Mean (SD) ^a^ or Median (IQR) ^b^		
Objective Nalmefene Knowledge Score	43.75 (37.50, 59.38)	50.00 (37.50, 62.50)	0.092 ^d^	0.315
Subjective Nalmefene Knowledge Scale	2.68 (0.91)	2.61 (0.76)	0.084 ^c^	0.675
Attitudes Toward Nalmefene Scale	3.72 (0.51)	3.67 (0.53)	0.090 ^c^	0.657
Clinical Attitudes Toward Nalmefene	3.75 (3.63, 4.13)	3.75 (3.50, 4.09)	0.048 ^d^	0.602
Interprofessional Recommendation Attitudes	3.50 (3.25, 4.00)	3.50 (3.00, 4.00)	0.128 ^d^	0.162
Subjective Norms Regarding Nalmefene Scale	2.67 (2.33, 3.33)	3.00 (2.50, 3.17)	0.100 ^d^	0.273
External to Workplace Subjective Norms	2.33 (1.67, 2.67)	2.50 (2.00, 3.00)	0.202 ^d^	0.027 *
Internal Workplace Subjective Norms	3.33 (3.00, 4.00)	3.33 (3.00, 3.67)	0.071 ^d^	0.438
Perceived Behavioral Control Scale	3.52 (0.83)	3.24 (0.72)	0.368 ^c^	0.073
Nalmefene Stocking and Procurement	3.83 (2.83, 4.50)	3.17 (2.67, 4.00)	0.195 ^d^	0.034 *
Nalmefene Counseling Confidence	3.60 (2.40, 4.00)	3.40 (2.55, 4.00)	0.063 ^d^	0.493
Barriers Regarding Nalmefene Scale	3.01 (0.48)	2.98 (0.55)	0.062 ^c^	0.769
Fiscal and Logistical Barriers	3.45 (0.66)	3.34 (0.62)	0.180 ^c^	0.390
Ethical and Stakeholder Barriers	2.51 (0.50)	2.56 (0.65)	0.075 ^c^	0.719
Nalmefene Services Intentions Scale	3.08 (2.92, 3.50)	3.00 (2.79, 3.50)	0.034 ^d^	0.710

^a^ *t*-test. ^b^ Mann–Whitney U test. ^c^ Cohen’s *d* = |*d*|. ^d^ Rank biserial correlation coefficient (*r*_rb_) = |Z|/√N; * *p* < 0.05.

**Table 4:** Comparison of inpatient and outpatient pharmacies (*n* = 119).

Scale	Pharmacy Inpatient–Outpatient Designation		Effect Size ^c,d^	*p*-Value
	Inpatient *n* = 25	Outpatient *n* = 94		
	Mean (SD) ^a^ or Median (IQR) ^b^	Mean (SD) ^a^ or Median (IQR) ^b^		
Objective Nalmefene Knowledge Score	50.00 (37.50, 62.50)	50.00 (37.00, 62.50)	0.061 ^d^	0.504
Subjective Nalmefene Knowledge Scale	2.96 (0.94)	2.55 (0.74)	0.519 ^c^	0.023 *
Attitudes Toward Nalmefene Scale	3.61 (0.71)	3.71 (0.47)	0.142 ^c^	0.538
Clinical Attitudes Toward Nalmefene	3.69 (3.25, 4.13)	3.75 (3.63, 4.13)	0.067 ^d^	0.464
Interprofessional Recommendation Attitudes	3.25 (2.75, 4.00)	3.50 (3.06, 4.00)	0.123 ^d^	0.178
Subjective Norms Regarding Nalmefene Scale	3.00 (2.54, 3.79)	2.83 (2.50, 3.00)	0.200 ^d^	0.029 *
External to Workplace Subjective Norms	3.00 (2.33, 3.58)	2.33 (1.67, 3.00)	0.264 ^d^	0.004 **
Internal Workplace Subjective Norms	3.50 (2.75, 4.00)	3.00 (3.00, 3.67)	0.107 ^d^	0.243
Perceived Behavioral Control Scale	3.37 (0.86)	3.31 (0.74)	0.080 ^c^	0.728
Nalmefene Stocking and Procurement	3.08 (2.67, 4.00)	3.24 (2.67, 4.00)	0.034 ^d^	0.712
Nalmefene Counseling Confidence	3.40 (2.70, 4.00)	3.40 (2.40, 4.00)	0.073 ^d^	0.425
Barriers Regarding Nalmefene Scale	2.75 (0.56)	3.05 (0.51)	0.567 ^c^	0.017 *
Fiscal and Logistical Barriers	3.07 (0.66)	3.45 (0.59)	0.628 ^c^	0.009 **
Ethical and Stakeholder Barriers	2.40 (0.63)	2.59 (0.60)	0.316 ^c^	0.181
Nalmefene Services Intentions Scale	3.08 (2.08, 3.50)	3.08 (2.92, 3.50)	0.063 ^d^	0.490

^a^ *t*-test. ^b^ Mann–Whitney U test. ^c^ Cohen’s *d* = |*d*|. ^d^ Rank biserial correlation coefficient (*r*_rb_) = |Z|/√N. ** *p* < 0.01, * *p* < 0.05.

**Table 5:** Comparison of chain and independent community pharmacies (*n* = 73).

Scale	Community Pharmacy Type		Effect Size ^c,d^	*p*-Value
	Chain *n* = 31	Independent *n* = 42		
	Mean (SD) ^a^ or Median (IQR) ^b^	Mean (SD) ^a^ or Median (IQR) ^b^		
Objective Nalmefene Knowledge Questions	50.00 (25.00, 62.50)	50.00 (37.50, 62.50)	0.090 ^d^	0.442
Subjective Nalmefene Knowledge Scale	2.60 (0.66)	2.48 (0.81)	0.159 ^c^	0.508
Attitudes Toward Nalmefene Scale	3.80 (0.49)	3.64 (0.47)	0.354 ^c^	0.144
Clinical Attitudes Toward Nalmefene	3.88 (3.63, 4.25)	3.69 (3.50, 3.91)	0.168 ^d^	0.152
Interprofessional Recommendation Attitudes	3.50 (3.25, 4.00)	3.50 (3.19, 3.81)	0.064 ^d^	0.586
Subjective Norms Regarding Nalmefene Scale	2.83 (2.50, 3.08)	2.83 (2.33, 3.04)	0.028 ^d^	0.809
External to Workplace Subjective Norms	2.33 (1.67, 2.50)	2.33 (1.67, 3.00)	0.010 ^d^	0.929
Internal Workplace Subjective Norms	3.33 (3.00, 3.67)	3.00 (3.00, 3.67)	0.096 ^d^	0.414
Perceived Behavioral Control Scale	3.24 (0.76)	3.48 (0.71)	0.338 ^c^	0.166
Nalmefene Stocking and Procurement	3.17 (2.67, 3.92)	3.75 (3.00, 4.17)	0.260 ^d^	0.026 *
Nalmefene Counseling Confidence	3.40 (2.70, 4.00)	3.40 (2.40, 4.00)	0.015 ^d^	0.897
Barriers Regarding Nalmefene Scale	3.01 (0.65)	3.09 (0.40)	0.158 ^c^	0.522
Fiscal and Logistical Barriers	3.37 (0.63)	3.57 (0.51)	0.349 ^c^	0.159
Ethical and Stakeholder Barriers	2.57(0.80)	2.55 (0.48)	0.027 ^c^	0.911
Nalmefene Services Intentions Scale	3.00 (2.88, 3.46)	3.08 (3.00, 3.56)	0.098 ^d^	0.401

^a^ *t*-test. ^b^ Mann-Whitney U test. ^c^ Cohen’s *d* = |*d*|. ^d^ Rank biserial correlation coefficient (*r*_rb_) = |Z|/√N. * *p* < 0.05.

**Table 6:** Comparison of OUD experience groups (*n* = 119).

Scale	Personally/Professionally Affected by OUD		Effect Size ^c,d^	*p*-Value
	No *n* = 69	Yes *n* = 50		
	Mean (SD) ^a^ or Median (IQR) ^b^	Mean (SD) ^a^ or Median (IQR) ^b^		
Objective Nalmefene Knowledge Questions	50.00 (37.50, 62.50)	50.00 (37.50, 62.50)	0.048 ^d^	0.604
Subjective Nalmefene Knowledge Scale	2.46 (0.73)	2.88 (0.85)	0.526 ^c^	0.006 **
Attitudes Toward Nalmefene Scale	3.67 (0.51)	3.72 (0.55)	0.482 ^c^	0.468
Clinical Attitudes Toward Nalmefene	3.75 (3.50, 4.00)	3.88 (3.63, 4.13)	0.074 ^d^	0.417
Interprofessional Recommendation Attitudes	3.50 (3.00, 4.00)	3.50 (3.00, 4.00)	0.053 ^d^	0.564
Subjective Norms Regarding Nalmefene Scale	3.00 (2.50, 3.00)	2.75 (2.46, 3.33)	0.020 ^d^	0.824
External to Workplace Subjective Norms	2.33 (1.67, 3.00)	2.33 (2.00, 3.00)	0.033 ^d^	0.721
Internal Workplace Subjective Norms	3.33 (3.00, 3.67)	3.00 (2.67, 4.00)	0.021 ^d^	0.823
Perceived Behavioral Control Scale	3.19 (0.73)	3.49 (0.77)	0.400 ^c^	0.037 *
Nalmefene Stocking and Procurement	3.17 (2.67, 4.00)	3.50 (2.83, 4.17)	0.138 ^d^	0.133
Nalmefene Counseling Confidence	3.40 (2.40, 3.90)	3.80 (2.90, 4.00)	0.192 ^d^	0.036 *
Barriers Regarding Nalmefene Scale	3.01 (0.46)	2.97 (0.62)	0.081 ^c^	0.675
Fiscal and Logistical Barriers	3.36 (0.53)	3.39 (0.73)	0.039 ^c^	0.838
Ethical and Stakeholder Barriers	2.60 (0.55)	2.49 (0.68)	0.174 ^c^	0.366
Nalmefene Services Intentions Scale	3.00 (2.92, 3.42)	3.08 (2.71, 3.36)	0.041 ^d^	0.657

^a^ *t*-test. ^b^ Mann–Whitney U test. ^c^ Cohen’s *d* = |*d*|. ^d^ Rank biserial correlation coefficient (*r*_rb_) = |Z|/√N. Abbreviations: OUD = opioid use disorder. ** *p* < 0.01, * *p* < 0.05.

**Table 7:** Comparison of managerial and non-managerial pharmacy positions (*n* = 119).

Scale	Pharmacy Position		Effect Size ^c,d^	*p*-Value
	Managerial *n* = 46	Non-Managerial *n* = 73		
	Mean (SD) ^a^ or Median (IQR) ^b^	Mean (SD) ^a^ or Median (IQR) ^b^		
Objective Nalmefene Knowledge Questions	50.00 (37.50, 62.50)	50.00 (37.50, 62.50)	0.002 ^d^	0.980
Subjective Nalmefene Knowledge Scale	2.67 (0.80)	2.61 (0.81)	0.074 ^c^	0.694
Attitudes Toward Nalmefene Scale	3.77 (0.45)	3.64 (0.57)	0.282 ^c^	0.143
Clinical Attitudes Toward Nalmefene	3.88 (3.63, 4.25)	3.75 (3.38, 4.09)	0.075 ^d^	0.414
Interprofessional Recommendation Attitudes	3.50 (3.50, 4.00) ^e^	3.50 (3.00, 4.00) ^e^	0.209 ^d^	0.023 *
Subjective Norms Regarding Nalmefene Scale	3.00 (2.50, 3.17)	2.83 (2.50, 3.00)	0.078 ^d^	0.395
External to Workplace Subjective Norms	2.33 (2.00, 3.00)	2.33 (1.67, 3.00)	0.017 ^d^	0.856
Internal Workplace Subjective Norms	3.67 (3.00, 4.00)	3.00 (3.00, 3.67)	0.140 ^d^	0.126
Perceived Behavioral Control Scale	3.63 (0.70)	3.14 (0.74)	0.670 ^c^	<0.001 ***
Nalmefene Stocking and Procurement	4.00 (3.17, 4.50)	3.00 (2.50, 3.67)	0.400 ^d^	<0.001 ***
Nalmefene Counseling Confidence	3.60 (2.80, 4.00)	3.40 (2.40, 4.00)	0.056 ^d^	0.544
Barriers Regarding Nalmefene Scale	2.89 (0.53)	3.06 (0.53)	0.319 ^c^	0.107
Fiscal and Logistical Barriers	3.31 (0.67)	3.42 (0.60)	0.176 ^c^	0.372
Ethical and Stakeholder Barriers	2.41 (0.55)	2.64 (0.64)	0.370 ^c^	0.061
Nalmefene Services Intentions Scale	3.00 (3.00, 3.42)	3.08 (2.58, 3.50)	0.030 ^d^	0.745

^a^ *t*-test. ^b^ Mann–Whitney U test. ^c^ Cohen’s *d* = |*d*|. ^d^ Rank biserial correlation coefficient (*r*_rb_) = |Z|/√N. ^e^ Mean (SD) values: managerial (3.65 [0.47]), non-managerial (3.39 [0.73]). *** *p* < 0.001, * *p* < 0.05.

**Table 8:** Association between Theory of Planned Behavior (TPB) constructs and the intention to implement nalmefene services in the next 6 months, full model (*n* = 119).

	Unadjusted ^a^			Adjusted for Sociodemographics ^b^		
Construct/Characteristic	B	95% CI	*p*-Value	B	95% CI	*p*-Value
Intercept	0.166	(−1.291, 1.623)	0.882	1.940	(−0.009, 3.889)	0.051
Objective Nalmefene Knowledge	0.001	(−0.005, 0.008)	0.682	0.000	(−0.007, 0.007)	0.960
Subjective Nalmefene Knowledge Scale	0.018	(−0.195, 0.232)	0.866	0.033	(−0.205, 0.271)	0.783
Attitudes: Clinical Attitudes	0.203	(−0.066, 0.473)	0.138	0.144	(−0.154, 0.442)	0.339
Attitudes: Interprofessional Recommendation	0.269	(0.057, 0.480)	0.013 *	0.169	(−0.060, 0.398)	0.145
SN: External to Workplace Subjective Norms	0.104	(−0.084, 0.292)	0.273	0.186	(−0.044, 0.417)	0.112
SN: Internal Workplace Subjective Norms	0.032	(−0.186, 0.250)	0.771	0.028	(−0.216, 0.272)	0.818
PBC: Nalmefene Stocking and Procurement	0.215	(0.063, 0.367)	0.006 **	0.170	(0.002, 0.338)	0.047 *
PBC: Nalmefene Counseling Confidence	0.023	(−0.180, 0.2226)	0.822	0.020	(−0.204, 0.244)	0.861
Barriers: Fiscal and Logistical Barriers	−0.160	(−0.392, 0.072)	0.175	−0.207	(−0.458, 0.045)	0.106
Barriers: Ethical and Stakeholder Barriers	0.169	(−0.105, 0.442)	0.224	−0.018	(−0.338, 0.302)	0.912
Age	-	-	-	0.002	(−0.009, 0.014)	0.671
Gender						
Male	-	-	-	−0.088	(−0.381, 0.205)	0.552
Is a Racial Minority						
No	-	-	-	−0.474	(−1.049, 0.102)	0.105
Has Experience with OUD						
No	-	-	-	0.029	(−0.237, 0.295)	0.831
Is a Manger/Administrator						
Yes	-	-	-	0.007	(−0.279, 0.294)	0.959
Pharmacy Rural-Urban Designation						
Urban	-	-	-	0.013	(−0.267, 0.294)	0.925
Pharmacy/Workplace Setting						
Inpatient	-	-	-	−0.358	(−0.718, −0.002)	0.048 *
Age of Patient Population						
Primarily Children/Adolescents	-	-	-	−0.091	(−1.389, 1.206)	0.889
Primarily Younger Adults (e.g., <65 y.o.)	-	-	-	0.149	(−0.470, 0.768)	0.634
Primarily Older Adults (e.g., >65 y.o.)	-	-	-	−0.269	(−0.602, 0.064)	0.112
Pharmacy Serves Indigent/Low-Income Patients						
No	-	-	-	−0.155	(−0.450, 0.140)	0.300

^a^ Unadjusted: F(10, 94) = 5.754, *p* < 0.001; R^2^ = 0.380; adj. R^2^ = 0.314. ^b^ Adjusted: F(21, 79) = 3.098, *p* < 0.001; R^2^ = 0.452; adj. R^2^ = 0.306. Diagnostics were acceptable for both unadjusted and adjusted models (VIF ≤ 3.43; tolerance ≥ 0.29; Durbin–Watson, 1.46–1.65; Cook’s D < 1); residual plots showed no substantial assumption violations. Abbreviations: B = unstandardized regression coefficient; Opioid Use Disorder (OUD). ** *p* < 0.01, * *p* < 0.05.

**Table 9:** Association between Theory of Planned Behavior (TPB) constructs and the intention to implement nalmefene services in the next 6 months, reduced model (*n* = 119).

	Unadjusted ^a^			Adjusted for Sociodemographics ^b^		
Construct/Characteristic	B	95% CI	*p*-Value	B	95% CI	*p*-Value
Intercept	0.450	(−0.813, 1.713)	0.481	1.205	(−0.165, 2.576)	0.084
Attitudes: Clinical Attitudes	0.209	(−0.037, 0.455)	0.094	0.172	(−0.071, 0.416)	0.164
Attitudes: Interprofessional Recommendation	0.279	(0.079, 0.478)	0.007 **	0.237	(0.037, 0.437)	0.021 *
SN: External to Workplace Subjective Norms	0.119	(−0.049, 0.286)	0.162	0.232	(0.047, 0.418)	0.015 *
PBC: Nalmefene Stocking and Procurement	0.217	(0.076, 0.357)	0.003 **	0.189	(0.049, 0.329)	0.009 **
Barriers: Fiscal and Logistical Barriers	−0.151	(−0.377, 0.074)	0.186	−0.146	(−0.371, 0.079)	0.201
Barriers: Ethical and Stakeholder Barriers	0.126	(−0.121, 0.372)	0.314	0.061	(−0.190, 0.312)	0.632
Is a Racial Minority						
No	-	-	-	−0.352	(−0.814, 0.111)	0.134
Pharmacy/Workplace Setting						
Inpatient	-	-	-	−0.366	(−0.668, −0.065)	0.018 *
Pharmacy Serves Indigent/Low-Income Patients						
No	-	-	-	−0.141	(−0.389, 0.108)	0.263

^a^ Unadjusted: F(6, 99) = 9.932, *p* < 0.001; R^2^ = 0.376; adj. R^2^ = 0.338. ^b^ Adjusted: F(9, 95) = 7.633, *p* < 0.001; R^2^ = 0.420; adj. R^2^ = 0.365. Diagnostics were acceptable for both unadjusted and adjusted models (VIF ≤ 2.10; tolerance ≥ 0.48; Durbin–Watson, 1.50–1.61; Cook’s D < 1); residual plots showed no substantial assumption violations. Abbreviations: B = unstandardized regression coefficient. ** *p* < 0.01, * *p* < 0.05.

## Data Availability

The data presented in this study are available on request from the corresponding author due to restrictions within the Institutional Review Board protocol.

## Supplementary Materials

The following supporting information can be downloaded at: https://www.mdpi.com/article/10.3390/pharmacy14060137/s1, File S1: Survey Instrument; File S2: Supplemental Tables.

## Abbreviations

The following abbreviations are used in this manuscript:

B	Unstandardized Regression Coefficient
FDA	Food and Drug Administration
MME	Morphine Milligram Equivalents
OUD	Opioid Use Disorder
PBC	Perceived Behavioral Control
SN	Subjective Norms
TPB	Theory of Planned Behavior
U.S.	United States
