# Supplementary material for: Knowledge and Beliefs Surrounding Nalmefene Among Alabama Pharmacists: A Cross-Sectional Survey Informed by the Theory of Planned Behavior
Source: Pharmacy (Basel). 2026 Sep 17;14(6):137. doi: 10.3390/pharmacy14060137 (PMC13610114; doi:10.3390/pharmacy14060137)
Supplement: Supplementary file 1 [file pharmacy-14-00137-s001.zip › Supplemental File S1_Survey Instrument.pdf]

Opvee Pharmacist Survey

Start of Block: Introduction

Q28
Pharmacist Knowledge and Perceptions Surrounding Nalmefene: A Cross-Sectional Survey in Alabama

Thank you for your participation in this project! The purpose of this research study is to better understand Alabama pharmacists' knowledge, beliefs, intentions, and current behaviors surrounding stocking, selling, and recommending nalmefene (Opvee®) to their patients in rural versus urban pharmacy settings. To that end, this survey will ask about your knowledge and opinions regarding nalmefene. This will help us develop education materials that are targeted to your needs and the needs of your pharmacy or workplace. This survey consists of 7 sections and should take about 20 minutes to complete. All responses will remain anonymous.

The Information Letter with more details regarding this research study can be reviewed here: INFORMATION LETTER

Clicking the "NEXT" button below serves as your consent to participate in this survey study.

End of Block: Introduction

Start of Block: Demographics

Q30 **I. INFORMATION ABOUT YOU**

Q1 Please select your gender.

- ○ Male
- ○ Female
- ○ Non-binary
- ○ Other. Please specify: ____
- ○ Prefer not to answer

Q2 How would you describe yourself? Please select all that apply.

- ☐ American Indian or Alaskan Native
- ☐ Asian
- ☐ Black or African American
- ☐ Latino(a)
- ☐ Native Hawaiian or Other Pacific Islander
- ☐ White
- ☐ Other. Please specify: ____

Q3 In which Alabama COUNTY is your pharmacy or workplace located, e.g. Jefferson County?

▼ Autauga ... Winston

Q4 What is your age in years?

____

Q5 What type of pharmacy or workplace do you practice in?

- ○ Community: Independently-owned pharmacy (e.g., Hometown Pharmacy)
- ○ Community: Corporately-owned pharmacy (e.g., CVS or Walgreen's)
- ○ Community: Grocery store pharmacy (e.g., Publix or Kroger)
- ○ Community: Big box store (e.g., Target, Walmart)
- ○ Hospital: Inpatient pharmacy
- ○ Hospital: Outpatient pharmacy
- ○ Clinic
- ○ Specialty pharmacy
- ○ Other. Please specify: ____

Q6 What is your position in the workplace?

- ○ Pharmacy manager
- ○ Assistant pharmacy manager
- ○ Full-time staff pharmacist
- ○ Part-time staff pharmacist
- ○ Floating pharmacist
- ○ Clinical pharmacist
- ○ Other. Please specify: ____

Q8 What type of patient population does your pharmacy or workplace serve? Please select all that apply.

- ▢ Primarily younger patients (e.g.
- ▢ Primarily older patients (e.g. >65)
- ▢ Primarily children and adolescents
- ▢ A mix of younger adults, older adults, and children
- ▢ Indigent and/or low income
- ▢ Other. Please specify: ____

Q9a Have you been personally affected by Opioid Use Disorder (OUD)?

- ○ Yes
- ○ No
- ○ Prefer not to answer

Q9b Have you been professionally affected by Opioid Use Disorder (OUD)?

- ○ Yes
- ○ No
- ○ Prefer not to answer

Q29 Before this survey, had you ever heard of nalmefene?

- ○ Yes
- ○ No
- ○ Unsure

Q3 Have you seen nalmefene in practice?

- ○ Yes
- ○ No
- ○ Unsure

Q10 In your professional capacity, have you ever been asked about nalmefene before?

○ Yes

○ No

Skip To: End of Block If In your professional capacity, have you ever been asked about nalmefene before? = No

---

Q11 Who asked you about nalmefene? Please select all that apply.

☐ Doctor

☐ Patient

☐ Caregiver

☐ Another pharmacist

☐ Law enforcement

☐ Other. Please specify:

____

End of Block: Demographics

Start of Block: Knowledge

Q31 **II. KNOWLEDGE ABOUT NALMEFENE** Please answer the following questions about nalmefene to the best of your ability. If you do not know the answer, please select "Unsure."

Q2 Nalmefene (Opvee) is used to _________.

- ○ Reverse opioid overdose
- ○ Reverse stimulant overdose
- ○ Manage symptoms of withdrawal from opioids
- ○ Manage symptoms of withdrawal from benzodiazepines
- ○ Unsure

Page Break

Nalmefene is an opioid antagonist indicated for the emergency treatment of known or suspected overdose induced by natural or synthetic opioids in adults and pediatric patients aged 12 years and older, as manifested by respiratory and/or central nervous system depression.

Page Break

Q6 How can nalmefene be administered?

- Nasal spray, intramuscular injection, subcutaneous injection, or intravenous injection
- Nasal spray only
- Intramuscular injection and subcutaneous injection
- Subcutaneous injection only
- Unsure

Page Break

Q32 The FDA approved the first prescription nalmefene hydrochloride nasal spray (Opvee) in May 2023 to reverse opioid overdose. As of August 2024, the FDA approved a nalmefene auto-injector formulation (Zurnai) for intramuscular or subcutaneous administration; Zurnai will be available on the market in 2025. A generic version of nalmefene is available in an injection vial for intravenous administration.

Page Break

Q4 What is the onset of action of nalmefene (Opvee) nasal spray?

- 1 minute
- 2-5 minutes
- 6 minutes
- 10-15 minutes
- Unsure

Q34 What is the duration of action of nalmefene (Opvee) nasal spray? Please select all that apply.

- As long as most opioids, including fentanyl
- Longer than naloxone (Narcan) nasal spray
- Shorter than naloxone (Narcan) nasal spray
- About one hour
- Unsure

Page Break

Q35 Nalmefene (Opvee) nasal spray has an onset of action of about 2-5 minutes. Its duration of action is as long as most opioids, including fentanyl, and is longer than naloxone (Narcan) nasal spray.

Page Break

Q5 What is the cost (wholesale acquisition cost) of nalmefene (Opvee) nasal spray?

- ◯ $37.50 per dose
- ◯ $49.00 per dose
- ◯ $210.00 per dose
- ◯ $21.99 per dose
- ◯ Unsure

Page Break

Qualifying direct purchasers, such as departments of health, emergency medical services, law enforcement, schools/universities, and community organizations, may be eligible to purchase nalmefene (OPVEE®) at the public interest price, which is $37.50/dose or $75/2-pack (as of October 2, 2023). For other orders, the Wholesale Acquisition Cost (WAC)* of OPVEE® is $49/dose or $98/2-pack (as of October 2, 2023)

Page Break

Subjective Knowledge Please rate your level of agreement or disagreement about the following statements regarding your knowledge of nalmefene.

	Strongly Disagree	Disagree	Neutral	Agree	Strongly Agree
I already have enough training about nalmefene.	○	○	○	○	○
I need more information about nalmefene.	○	○	○	○	○
I know how to treat an acute opioid overdose event.	○	○	○	○	○
I know where to seek out training about nalmefene.	○	○	○	○	○
I know how much nalmefene costs.	○	○	○	○	○
I know how to counsel a patient about nalmefene.	○	○	○	○	○
I know the differences between nalmefene and naloxone.	○	○	○	○	○

End of Block: Knowledge

Start of Block: Attitudes

Q36 **III. ATTITUDES ABOUT NALMEFENE**

Q30 Please rate your level of agreement or disagreement about the following statements regarding your attitudes surrounding nalmefene.

	Strongly Disagree	Disagree	Neutral	Agree	Strongly Agree
Stocking nalmefene in my workplace would be worth my time	○	○	○	○	○
It is important for pharmacists to be knowledgable about nalmefene	○	○	○	○	○
Nalmefene is a good option for patients at risk of opioid overdose	○	○	○	○	○
I would recommend nalmefene to a patient	○	○	○	○	○
I would recommend nalmefene to a patient instead of naloxone	○	○	○	○	○
Nalmefene is a valuable addition to the options for opioid overdose reversal	○	○	○	○	○
Since naloxone is already on the market, nalmefene is not needed	○	○	○	○	○

Pharmacists should dispense nalmefene by prescription only	○	○	○	○	○
Similar to naloxone, nalmefene should be dispensed by pharmacists via standing order	○	○	○	○	○
Pharmacists have the education and training to educate patients on how to recognize the signs of opioid overdose and its emergency management	○	○	○	○	○
It is appropriate for pharmacists to dispense nalmefene to prevent opioid overdose	○	○	○	○	○
I would recommend nalmefene to another healthcare provider	○	○	○	○	○
I would recommend nalmefene instead of	○	○	○	○	○

naloxone to another healthcare provider					
I would recommend nalmefene to a law enforcement officer	○	○	○	○	○
I would recommend nalmefene instead of naloxone to a law enforcement officer	○	○	○	○	○

End of Block: Attitudes

Start of Block: Subjective Norms

Q37 **IV. COMMUNITY AND WORKPLACE PERCEPTIONS OF NALMEFENE**

Q31 Please rate your level of agreement or disagreement about the following statements regarding your perceptions of how nalmefene is viewed in your community or workplace.

	Strongly Disagree	Disagree	Neutral	Agree	Strongly Agree
Nalmefene is being prescribed by providers in my area	○	○	○	○	○
Nalmefene is being used in my community	○	○	○	○	○
My patients are open to using nalmefene	○	○	○	○	○
My coworkers are open to recommending nalmefene	○	○	○	○	○
The person overseeing inventory in my workplace would be open to stocking nalmefene	○	○	○	○	○
Coworkers would support me recommending nalmefene to patients	○	○	○	○	○

End of Block: Subjective Norms

Start of Block: Perceived Behavioral Control

Q38 **V. COMFORT AND CONFIDENCE WITH STOCKING AND RECOMMENDING NALMEFENE**

Q32 Please rate your level of agreement or disagreement about the following statements regarding your comfort and confidence with stocking and recommending nalmefene.

	Strongly Disagree	Disagree	Neutral	Agree	Strongly Agree
I have no control over whether nalmefene is stocked in my workplace	○	○	○	○	○
It is not my decision about stocking nalmefene in my workplace	○	○	○	○	○
If I decide to stock nalmefene in my workplace, I can do so easily	○	○	○	○	○
I feel comfortable recommending stocking nalmefene to my manager	○	○	○	○	○
I feel comfortable suggesting nalmefene to the person who orders stock in my workplace	○	○	○	○	○
I feel comfortable ordering nalmefene myself to stock in the workplace	○	○	○	○	○
I feel confident in my ability to counsel	○	○	○	○	○

patients on nalmefene					
I feel confident in my ability to counsel other healthcare providers on nalmefene	○	○	○	○	○
I feel confident in my ability to counsel law enforcement officers on nalmefene	○	○	○	○	○
I feel confident identifying individuals at risk of experiencing an opioid-related overdose who could benefit from nalmefene	○	○	○	○	○
I would feel confident communicating with patients about the need for nalmefene	○	○	○	○	○

End of Block: Perceived Behavioral Control

Start of Block: Perceived Barriers

Q39 **VI. PERCEIVED BARRIERS TO STOCKING AND PROVIDING NALMEFENE**

Q27 Please rate your level of agreement or disagreement about the following statements regarding barriers to stocking and providing nalmefene.

	Strongly Disagree	Disagree	Neutral	Agree	Strongly Agree
The cost of stocking nalmefene products is a concern in my workplace	○	○	○	○	○
I do not have a physician partner to authorize a protocol to dispense nalmefene	○	○	○	○	○
Physicians in my area will not prescribe nalmefene	○	○	○	○	○
I have moral/ethical concerns with dispensing nalmefene	○	○	○	○	○
Reimbursement associated with dispensing nalmefene is a concern in my workplace	○	○	○	○	○
There is a lack of knowledge/training about nalmefene among staff in my workplace	○	○	○	○	○
There is a lack of knowledge/training about nalmefene among local prescribers	○	○	○	○	○
There is a lack of support from my manager to provide nalmefene	○	○	○	○	○

There will be an increased number of people who misuse opioids who visit my workplace if we provide nalmefene	○	○	○	○	○
A nalmefene dispensing program would be difficult to incorporate into pharmacy workflow	○	○	○	○	○
It would be too time-consuming to provide nalmefene at my workplace	○	○	○	○	○
My patients will not be able to afford nalmefene	○	○	○	○	○
Nalmefene may require a prior authorization from insurance companies	○	○	○	○	○
Naloxone will remain the priority to stock in the pharmacy	○	○	○	○	○
The cost of stocking both nalmefene and naloxone is a concern	○	○	○	○	○
Since naloxone is now over-the-counter, patient demand for prescription items like nalmefene will decline	○	○	○	○	○
Patients will not be interested in nalmefene	○	○	○	○	○

I do not know how to approach patients about the need for nalmefene	○	○	○	○	○
Patients will be angry if I talk to them about nalmefene	○	○	○	○	○

End of Block: Perceived Barriers

Start of Block: Intentions

Q40 **VII. INTENTIONS TO PROVIDE NALMEFENE**

Q28 Please indicate how likely you are to do the following: ***"In the next 6 months, I intend to..."***

	Strongly Disagree	Disagree	Neutral	Agree	Strongly Agree
Stock nalmefene in my workplace	○	○	○	○	○
Counsel patients on how to use nalmefene in case of an opioid overdose	○	○	○	○	○
Recommend nalmefene to patients prescribed opioids for treatment of pain	○	○	○	○	○
Recommend nalmefene to patients suspected of using illicit opioids	○	○	○	○	○
Recommend nalmefene to patients using highly potent opioids, like fentanyl	○	○	○	○	○
Recommend nalmefene to patients prescribed opioids greater than or equal to 50 morphine milligram equivalents (MMEs) per day	○	○	○	○	○

Recommend nalmefene to law enforcement	○	○	○	○	○
Sell nalmefene in my workplace	○	○	○	○	○
Recommend nalmefene to other healthcare providers	○	○	○	○	○
Monitor patient outcomes after nalmefene administration	○	○	○	○	○
Prioritize ordering nalmefene when restocking my workplace	○	○	○	○	○
Stock both nalmefene and naloxone	○	○	○	○	○
Stock nalmefene instead of naloxone	○	○	○	○	○

End of Block: Intentions
