# Supplementary material for: Knowledge and Beliefs Surrounding Nalmefene Among Alabama Pharmacists: A Cross-Sectional Survey Informed by the Theory of Planned Behavior
Source: Pharmacy (Basel). 2026 Sep 17;14(6):137. doi: 10.3390/pharmacy14060137 (PMC13610114; doi:10.3390/pharmacy14060137)
Supplement: Supplementary file 1 [file pharmacy-14-00137-s001.zip › Supplemental File S2_Supplemental Tables_final.pdf]

Supplemental Table S1. Pharmacists' Objective Knowledge Regarding Nalmefene (N=119)

**Questions**	**n (%)**
Nalmefene (Opvee) is used to _________.	
*Reverse opioid overdose	79 (66.9)
Reverse stimulant overdose	-
Manage symptoms of withdrawal from opioids	8 (6.8)
Manage symptoms of withdrawal from benzodiazepines	-
Unsure	31 (26.3)
How can nalmefene be administered?	
*Nasal spray, intramuscular injection, subcutaneous injection, or intravenous injection	29 (24.6)
Nasal spray only	40 (33.9)
Intramuscular injection and subcutaneous injection	8 (6.8)
Subcutaneous injection only	-
Unsure	41 (34.7)
What is the onset of action of nalmefene (Opvee) nasal spray?	
1 minute	36 (30.3)
*2-5 minutes	35 (29.4)
6 minutes	1 (0.8)
10-15 minutes	2 (1.7)
Unsure	45 (37.8)
What is the duration of action of nalmefene (Opvee) nasal spray? Please select all that apply.	
*As long as most opioids, including fentanyl	13 (10.9)
*Longer than naloxone (Narcan) nasal spray	58 (48.7)
Shorter than naloxone (Narcan) nasal spray	5 (4.2)
About one hour	3 (2.5)
Unsure	54 (45.4)
What is the cost (wholesale acquisition cost) of nalmefene (Opvee) nasal spray?	
$37.50 per dose	10 (8.4)
*$49.00 per dose	26 (21.8)
$210.00 per dose	9 (7.6)
$21.99 per dose	3 (2.5)
Unsure	71 (59.7)

* Correct response

Supplemental Table S2. Pharmacists' Subjective Nalmefene Knowledge Scale Exploratory Factor Analysis (EFA) at Factor Loading Level of ≥ 0.40 (α = .798)

**Item Number**[†]	**n (%)**					**Component**
	**1**	**2**	**3**	**4**	**5**	**1**
**Subjective Nalmefene Knowledge (α = .798; EV = 3.087[a])**						
(1) I already have enough training about nalmefene	40 (33.6)	50 (42)	16 (13.4)	11 (9.2)	2 (1.7)	.758
(2) I need more information about nalmefene[‡]	1 (.8)	9 (7.6)	13 (10.9)	56 (47.1)	40 (33.6)	.532

(4) I know where to seek out training about nalmefene	10 (8.4)	21 (17.6)	31 (26.1)	39 (32.8)	18 (15.1)	.521
(6) I know how to counsel a patient about nalmefene	16 (13.4)	37 (31.1)	25 (21)	34 (28.6)	7 (5.9)	.815
(7) I know the differences between nalmefene and naloxone	9 (7.6)	36 (30.5)	22 (18.6)	41 (34.7)	10 (8.5)	.740

1=strongly disagree, 2=disagree, 3=neutral, 4=agree, 5=strongly agree

[a] Eigenvalue

† Two items, "(3) I know how to treat an acute opioid overdose event" and "(5) I know how much nalmefene costs were dropped due to failing to load at a factor loading level < .40.

‡ Indicates a reverse-coded Likert-type item.

Bartlett's Test of Sphericity: $x^2(21, n = 118) = 223.851$ ($p < 0.001$); Kaiser-Meyer-Olkin MSA = 0.791.

Supplemental Table S3. Pharmacists' Attitudes Toward Nalmefene Scale Exploratory Factor Analysis (EFA) at Factor Loading Level of ≥ 0.40 (α = .891)

**Item Number†**	**n (%)**					**Components**	
	**1**	**2**	**3**	**4**	**5**	**1**	**2**
**Positive Clinical Attitudes Toward Nalmefene (α = .857; EV = 5.887[a])**							
(1) Stocking nalmefene in my workplace would be worth my time	6 (5.1)	17 (14.5)	44 (37.6)	42 (35.9)	8 (6.8)	.430	
(2) It is important for pharmacists to be knowledgeable about nalmefene	1 (.9)		4 (3.4)	63 (53.8)	49 (41.9)	.718	
(3) Nalmefene is a good option for patients at risk of opioid overdose	2 (1.7)		18 (15.4)	64 (54.7)	33 (28.2)	.828	
(4) I would recommend nalmefene to a patient	2 (1.7)		37 (31.6)	59 (50.4)	19 (16.2)	.796	
(5) I would recommend nalmefene to a patient instead of naloxone	2 (1.7)	15 (12.8)	69 (59)	22 (18.8)	9 (7.7)	.517	
(6) Nalmefene is a valuable addition to the options for opioid overdose reversal	1 (.9)		15 (12.8)	73 (62.4)	28 (23.9)	.793	
(9) Similar to naloxone, nalmefene should be dispensed by pharmacists via standing order	1 (.9)	8 (6.8)	36 (30.8)	55 (47)	17 (14.5)	.457	
(11) It is appropriate for pharmacists to dispense nalmefene to prevent opioid overdose	2 (1.7)	2 (1.7)	25 (21.4)	64 (54.7)	24 (20.5)	.615	
**Interprofessional Recommendation Attitudes (α = .877; EV = 1.881[a])**							
(12) I would recommend nalmefene to another healthcare provider		5 (4.3)	28 (23.9)	64 (54.7)	20 (17.1)	.569	-.405
(13) I would recommend nalmefene instead of naloxone to another healthcare provider	2 (1.7)	13 (11.1)	70 (59.8)	26 (22.2)	6 (5.1)		-.876
14) I would recommend nalmefene to a law		5	40	56	16		-.622

enforcement officer		(4.3)	(34.2)	(47.9)	(13.7)	
(15) I would recommend nalmefene instead of naloxone to a law enforcement officer	1 (.9)	14 (12.1)	72 (62.1)	19 (16.4)	10 (8.6)	-.959

1=strongly disagree, 2=disagree, 3=neutral, 4=agree, 5=strongly agree
[a] Eigenvalue
† Three items, "(7) Since naloxone is already on the market, nalmefene is not needed," (8) Pharmacists should dispense nalmefene by prescription only," and "(10) Pharmacists have the education and training to educate patients on how to recognize the signs of opioid overdose and its emergency management" were dropped due to failing to load at a factor loading level ≥ .40.
Bartlett's Test of Sphericity: $x^2(91, n = 116) = 912.968$ ($p < 0.001$); Kaiser-Meyer-Olkin MSA = 0.842.

Supplemental Table S4. Pharmacists' Subjective Nalmefene Norms (SN) Scale Exploratory Factor Analysis (EFA) at Factor Loading Level of ≥ 0.40 (α = .785)

**Item Number†**	**n (%)**					**Components**	
	**1**	**2**	**3**	**4**	**5**	**1**	**2**
**External to Workplace Subjective Norms (α = .817; EV = 3.053[a])**							
(1) Nalmefene is being prescribed by providers in my area	35 (30.2)	40 (34.5)	31 (26.7)	8 (6.9)	2 (1.7)	-.942	
(2) Nalmefene is being used in my community	27 (23.3)	39 (33.6)	33 (33.6)	9 (7.8)	2 (1.7)	-.883	
(3) My patients are open to using nalmefene	5 (4.3)	8 (6.9)	87 (75)	15 (12.9)	1 (.9)	-.423	.502
**Internal Workplace Subjective Norms (α = .816; EV = 1.663[a])**							
(4) My coworkers are open to recommending nalmefene	3 (2.6)	12 (10.3)	72 (62.1)	27 (23.3)	2 (1.7)		.637
(5) The person overseeing inventory in my workplace would be open to stocking nalmefene	7 (6)	18 (15.5)	38 (32.8)	48 (41.4)	5 (4.3)		.841
(6) Coworkers would support me recommending nalmefene to patients	4 (3.4)	2 (1.7)	56 (48.3)	47 (40.5)	7 (6)		.890

1=strongly disagree, 2=disagree, 3=neutral, 4=agree, 5=strongly agree
[a] Eigenvalue
Bartlett's Test of Sphericity: $x^2(15, n = 116) = 356.271$ ($p < 0.001$); Kaiser-Meyer-Olkin MSA = 0.734.

Supplemental Table S5. Pharmacists' Perceived Behavioral Control (PBC) Scale Exploratory Factor Analysis (EFA) at Factor Loading Level of ≥ 0.40 (α = .883)

**Item Number†**	**n (%)**					**Components**	
	**1**	**2**	**3**	**4**	**5**	**1**	**2**

**Nalmefene Stocking and Procurement (α = .887; EV= 5.270[a])**							
(1) I have no control over whether nalmefene is stocked in my workplace[‡]	24 (21.1)	33 (28.9)	19 (16.7)	24 (21.1)	14 (12.2)	.798	
(2) It is not my decision about stocking nalmefene in my workplace[‡]	21 (18.4)	31 (27.2)	16 (14)	29 (25.4)	17 (14.9)	.871	
(3) If I decide to stock nalmefene in my workplace, I can do so easily	9 (7.9)	14 (12.3)	31 (27.2)	40 (35.1)	20 (17.5)	.805	
(4) I feel comfortable recommending stocking nalmefene to my manager	2 (1.8)	14 (12.3)	37 (32.5)	45 (39.5)	16 (14)	.549	
(5) I feel comfortable suggesting nalmefene to the person who orders stock in my workplace	4 (3.5)	13 (11.4)	34 (29.8)	45 (39.5)	18 (15.8)	.613	
(6) I feel comfortable ordering nalmefene myself to stock in the workplace	7 (6.1)	20 (17.5)	36 (31.6)	35 (30.7)	16 (14)	.760	
**Nalmefene Counseling Confidence (α = .915, EV = 2.575[a])**							
(7) I feel confident in my ability to counsel patients on nalmefene	7 (6.1)	24 (21.1)	30 (26.3)	43 (37.7)	10 (8.8)		.910
(8) I feel confident in my ability to counsel other healthcare providers on nalmefene	8 (7)	29 (25.4)	29 (25.5)	38 (33.3)	10 (8.8)		.972
(9) I feel confident in my ability to counsel law enforcement officers on nalmefene	8 (7)	27 (23.7)	28 (24.6)	42 (36.8)	9 (7.9)		.978
(10) I feel confident identifying individuals at risk of experiencing an opioid-related overdose who could benefit from nalmefene	4 (3.5)	18 (15.8)	22 (19.3)	59 (51.8)	11 (9.6)		.606
(11) I would feel confident communicating with patients about the need for nalmefene	6 (5.3)	19 (16.7)	15 (13.2)	61 (53.5)	13 (11.4)		.620

1=strongly disagree, 2=disagree, 3=neutral, 4=agree, 5=strongly agree

[a] Eigenvalue

[‡] Indicates a reverse-coded Likert-type item.

Bartlett's Test of Sphericity: $x^2(55, n = 114) = 1225.274$ ($p < 0.001$); Kaiser-Meyer-Olkin MSA = 0.822.

Supplemental Table S6. Pharmacists' Perceived Nalmefene Service Barriers Scale Exploratory Factor Analysis (EFA) at Factor Loading Level of ≥ 0.40 (α = .869)

Item Number[†]	n (%)					Components	
	1	2	3	4	5	1	2
**Fiscal and Logistical Barriers (α = .831; EV= 5.613[a])**							
(1) The cost of stocking nalmefene products is a concern in my workplace	4 (3.6)	27 (24.3)	30 (27)	39 (35.1)	11 (9.9)	.449	
(5) Reimbursement associated with dispensing nalmefene is a concern in my workplace	7 (6.3)	16 (14.4)	38 (34.2)	31 (27.9)	19 (17.1)	.677	
(6) There is a lack of knowledge/training about nalmefene among staff in my workplace	1 (.9)	12 (10.8)	18 (16.2)	57 (51.4)	23 (20.7)	.538	
(7) There is a lack of knowledge/training about nalmefene among local prescribers	1 (.9)	5 (4.5)	32 (29.1)	50 (45.5)	22 (20)	.564	
(12) My patients will not be able to afford nalmefene	6 (5.4)	12 (10.8)	44 (39.6)	38 (34.2)	11 (9.9)	.539	
(13) Nalmefene may require a prior authorization from insurance companies	1 (.9)	7 (6.3)	36 (32.4)	45 (40.5)	22 (19.8)	.676	
(15) The cost of stocking both nalmefene and naloxone is a concern	5 (4.5)	23 (20.9)	39 (35.5)	31 (28.2)	12 (10.9)	.574	
(16) Since naloxone is now over-the-counter, patient demand for prescription items like nalmefene will decline	6 (5.4)	24 (21.6)	42 (37.8)	33 (29.7)	6 (5.4)	.405	

(17) Patients will not be interested in nalmefene	2 (1.8)	33 (29.7)	60 (54.1)	13 (11.7)	3 (2.7)	.443
**Ethical and Stakeholder Barriers (α = .817, EV = 2.040[a])**						
(3) Physicians in my area will not prescribe nalmefene	5 (4.5)	18 (6.2)	77 (69.4)	6 (5.4)	5 (4.5)	.432
(4) I have moral/ethical concerns with dispensing nalmefene	34 (30.6)	45 (40.5)	27 (24.3)	3 (2.7)	2 (1.8)	.744
(8) There is a lack of support from my manager to provide nalmefene	13 (11.7)	33 (29.7)	46 (41.4)	17 (15.3)	2 (1.8)	.466
(9) There will be an increased number of people who misuse opioids who visit my workplace if we provide nalmefene	33 (29.7)	44 (39.6)	25 (22.5)	7 (6.3)	2 (1.8)	.580
(10) A nalmefene dispensing program would be difficult to incorporate into pharmacy workflow	8 (7.2)	30 (27)	41 (36.9)	28 (25.2)	4 (3.6)	.578
(11) It would be too time-consuming to provide nalmefene at my workplace	16 (14.4)	44 (39.6)	38 (34.2)	10 (9)	3 (2.7)	.848
(18) I do not know how to approach patients about the need for nalmefene	6 (5.4)	45 (40.5)	34 (30.6)	22 (19.8)	4 (3.6)	.486
(19) Patients will be angry if I talk to them about nalmefene	13 (11.8)	37 (33.6)	43 (39.1)	13 (11.8)	4 (3.6)	.477

1=strongly disagree, 2=disagree, 3=neutral, 4=agree, 5=strongly agree
[a] Eigenvalue
† Two items, "(2) I do not have a physician partner to authorize a protocol to dispense nalmefene" and "(14) Naloxone will remain the priority to stock in the pharmacy" were dropped due to failing to load at a factor loading level ≥ .40.
Bartlett's Test of Sphericity: $x^2(136, n = 109) = 741.893$ ($p < 0.001$); Kaiser-Meyer-Olkin MSA = 0.802.

Supplemental Table S7. Pharmacists' Reported Nalmefene Use Intentions Scale Exploratory Factor Analysis (EFA) at Factor Loading Level of ≥ 0.40 (α = .950)

**Item Number†**	**n (%)**					**Component**
	**1**	**2**	**3**	**4**	**5**	**1**
**Nalmefene Use Intentions (α = .950;**						

**EV= 7.831ᵃ)**						
(1) Stock nalmefene in my workplace	7 (6.4)	24 (22)	59 (54.1)	14 (12.8)	5 (4.6)	.736
(2) Counsel patients on how to use nalmefene in case of an opioid overdose	7 (6.4)	15 (13.8)	52 (47.7)	32 (29.4)	3 (2.8)	.813
(3) Recommend nalmefene to patients prescribed opioids for treatment of pain	5 (4.6)	17 (15.6)	56 (51.4)	28 (25.7)	3 (2.8)	.809
(4) Recommend nalmefene to patients suspected of using illicit opioids	7 (6.4)	17 (15.6)	53 (48.6)	30 (27.5)	2 (1.8)	.831
(5) Recommend nalmefene to patients using highly potent opioids, like fentanyl	5 (4.6)	15 (13.8)	42 (38.5)	43 (39.4)	4 (3.7)	.867
(6) Recommend nalmefene to patients prescribed opioids greater than or equal to 50 morphine milligram equivalents (MMEs) per day	5 (4.6)	13 (11.9)	50 (45.9)	39 (35.8)	2 (1.8)	.863
(7) Recommend nalmefene to law enforcement	4 (3.7)	15 (13.8)	57 (52.3)	31 (28.4)	2 (1.8)	.810
(8) Sell nalmefene in my workplace	8 (7.3)	19 (17.4)	64 (58.7)	15 (13.8)	3 (2.8)	.834
(9) Recommend nalmefene to other healthcare providers	3 (2.8)	12 (11)	55 (50.5)	33 (30.3)	6 (5.5)	.815
(10) Monitor patient outcomes after nalmefene administration	7 (6.4)	17 (15.6)	53 (48.6)	26 (23.9)	6 (5.5)	.612
(11) Prioritize ordering nalmefene when restocking my workplace	5 (4.6)	19 (17.4)	68 (62.4)	15 (13.8)	2 (1.8)	.774
(12) Stock both nalmefene and naloxone	5 (4.6)	14 (12.8)	63 (57.8)	21 (19.3)	6 (5.5)	.662

1=strongly disagree, 2=disagree, 3=neutral, 4=agree, 5=strongly agree

[a] Eigenvalue

† One item, “(13) Stock nalmefene instead of naloxone” was dropped due to failing to load at a factor loading level ≥ .40.

Bartlett’s Test of Sphericity: $x^2(66, n = 109) = 1231.808$ ($p < 0.001$); Kaiser-Meyer-Olkin MSA = 0.893.
